# Programmed wound healing in aged skin may be enhanced by mesenchymal cell loaded gene-activated scaffolds

**DOI:** 10.1063/5.0240504

**Published:** 2025-04-25

**Authors:** Priya Das, Martin Maresch, Nigamananda Dey, Noof Sulaiman, Amr Gamal Ashour, Hamad M. Ammar, Mohammed Basem, Mohammed A. Al Muharraqi, Matthew McGrath, Melvin Varghese Jacob, Fergal J. O'Brien, Michael B. Keogh

**Affiliations:** 1 TERG Bahrain, School of Postgraduate Studies and Research, Royal College of Surgeons in Ireland, Manama, Kingdom of Bahrain; 2 RMS Royal Medical Services, Riffa, Kingdom of Bahrain; 3 Tissue Engineering Research Group, Department of Anatomy, Royal College of Surgeons in Ireland, Dublin, Ireland; 4 Charis Veterinary Clinic, Budaiya, Kingdom of Bahrain; 5 Advanced Materials and Bioengineering Research Centre (AMBER), RCSI, Dublin, Ireland

## Abstract

Aging can prolong the wound healing and is associated with decline in stem cells, delays in cellular migration, and lower vascularization. Tissue engineering has largely evolved to incorporate advanced three-dimensional wound dressings, scaffolds, and hydrogels that may be seeded with mesenchymal stromal cells (MSCs) to foster an environment conducive to regeneration and enhance the healing process. The effectiveness of stem cell-seeded scaffolds can be improved by incorporating activating agents such as nucleic acids resulting in gene-activated scaffolds (GAS), thereby facilitating targeted wound healing in aged patients. In this study, we assess the *in vivo* wound healing potential of a promising MSC seeded gene-activated collagen scaffold, containing the anti-fibrotic agent β-klotho and pro-angiogenic stromal derived factor (SDF-1α) in aged male Sprague Dawley rats (20–24 month old). A MSC cell loaded split skin model compared MSC only with the clinical standard dressing +Jelonet, MSCs +gene-free collagen scaffold, and MSCs +SDF-1α/β-klotho dual gene-activated collagen scaffold up to 21 days. Our results showed wound healing in all groups except in MSC +Jelonet which showed scab formation with exudate. MSC only group healed primarily via fibrotic contraction. In contrast, the scaffold groups showed host tissue integration and a redistribution of extracellular matrix proteins, less contraction, and complete re-epithelized wounds at day 21. The dual GAS displayed programmed wound healing with the greatest neo-vascularization CD31 expression. In conclusion, wound healing in aged rats can be effectively modulated when MSCs are loaded on biocompatible collagen scaffolds, particularly when these scaffolds are loaded with anti-fibrotic and pro-angiogenic factors. This approach enhances blood vessel formation while reducing fibrosis, suggesting a promising potential for programmed wound healing strategies in aged chronic wounds.

## I. INTRODUCTION

Chronic wounds developed due to underlying clinical conditions such as diabetes, vascular diseases, aging, and genetic disorders (hemoglobinopathies) continue to be a medical burden and are a major cause of morbidity.^1,2^ Chronic wounds involve tissue damage to the deeper vasculodermal layer of the skin and pose a challenge to wound healing process.^3,4^ In addition, it is well established that aging further worsens the chronic wound healing process by prolonging the inflammatory phase, delayed monocyte influx, increased number of mature macrophages, and diminished cell migration and proliferation.^5,6^ The orderly sequence of four overlapping phases of tissue repair, including coagulation, inflammation, proliferation, and remodeling, is impaired in chronic wounds during aging, in diabetic and non-diabetic foot ulcers.^4,6^ In addition, it is well established that aging further worsens the chronic wound healing process by prolonging the inflammatory phase, delayed cellular response of stem cells, and macrophages due to age-related diminished cell migration and proliferation. This can impair neurogenic and angiogenic responses critical for wound healing.^5,6^ The rate of wound complications, lower extremity amputations, and ulcer reoccurrence is reportedly high in elderly patients.^7^ Aging is associated with delays and aberrations in macrophage and immune cell infiltration, vascularization, and wound modeling transfection.^7–10^ Despite this, no new therapy has gained Federal Drug Administration-approval for its treatment in decades, largely due to preclinical research and clinical trial challenges.^11^

The use of xenograft, dermal substitutes/bioengineered skin (Matriderm, Integra, Alloderm), and cellular dermal allograft (Dermagraft, Apligraf) are other alternative treatments being developed to address chronic wound healing and are only moderately effective and do not balance the cost involved.^12,13^ Standard skin ulcer wound care involves wound debridement followed by application of sterile gauze dressing like Suprathel^®^ or Jelonet^®^. Jelonet is a nonadherent paraffin coated gauze, which is most used dressing in burns units and widely used as a substitute for split skin grafts as an occlusive dressing. It keeps the wound moist and is often chosen as an inexpensive approach to keep the wound hydrated.^14^

One of the main concerns in treating a chronic wound in aged patients is that extreme inflammatory milieu turns the stem cells and endothelial cells into a senescent stage, a state of irreversible cell cycle arrest, which mitigates angiogenesis.^15^ Neo-vascularization in a wound is critical as it ensures the delivery of nutrients and oxygen.^16^ The senescence of these critical cell types in aging limits wound healing.^17^ To address these challenges, we have focused on the use of collagen–glycosaminoglycan scaffolds. These scaffolds offer several advantages, including high biocompatibility, which ensures minimal immune response, and porosity, which facilitates cell infiltration and nutrient exchange. These properties make the collagen–glycosaminoglycan scaffolds an optimum three-dimensional natural dermal substitute.^18,19^ These scaffolds have been successfully employed in the regeneration of bone, cartilage, and skin,^20–22^ demonstrating enhanced granulation tissue formation, angiogenesis, and reepithelization.^23–26^ However, there is no gold standard nor biomaterial specifically designed to treat a wound of an aged patient or premature aging like diabetic foot ulcer (DFU).^7^

Skin-resident stem cells play a major role in the orchestrated migration of endothelial cells, fibroblasts, keratinocytes, and melanocytes by enhancing the upregulation of chemokines like Insulin-like growth factor, Vascular endothelial growth factor, Transforming growth factor and SDF. In aged wounds, however, these pathways are often impaired.^16,27–29^ Mesenchymal stromal cells (MSCs) are excellent candidates for regenerative medicine and in treatment of chronic wounds owing to their multipotent and self-renewing properties. Similarly, bone marrow derived MSCs have broad differentiation potential, with reports of them being differentiated into keratinocytes, fibroblasts, and endothelial cells, additionally releasing factors enhancing cellular migration and cutaneous regeneration.^30^ They mitigate the pro-inflammatory effects of TNF-α and enhance the myofibroblast action to wound contraction through TGF-β1 mediated pathways.^31–33^ Even use of MSC conditioned media has emerged as a potential candidate for wound healing therapy, highlighting the paracrine activity of MSCs.^29,34,35^ Cellular grafts with MSCs alone or co-cultured endothelial cells have been reported to enhance angiogenesis and promote tissue repair and regeneration and may be supported with tissue engineering approaches of porous scaffolds loaded with pro-angiogenic activating molecules.^27,28^ It has been shown that novel cell-seeded biomaterials could stimulate allogenic stem cells to initiate wound healing.^36,37^ Additionally, pre-vascularized scaffolds/grafts are more likely to integrate in the wound.^38^ The innovative combination of tissue engineering and gene therapy, known as gene-activated scaffold (GAS), is emerging as a promising approach for restoring tissue function. These GAS could serve as a reservoir for functional genes, enabling *in situ* gene transfection and the expression of specific bio signals to modulate cellular behavior.^39^ Previously, our group has shown *in vitro* pro-angiogenic, anti-fibrotic, and wound healing ability of gene-activated collagen scaffolds (using plasmids for pro-angiogenic stromal derived factor-1α (SDF-1α) and anti-aging β-klotho and non-viral methods of gene transfection) seeded with mesenchymal stem cells.^40,41^ SDF-1α, a chemokine also known as CXCL12, binds to CXCR4 (G protein-coupled receptor present on various cells including endothelial cells), and this signaling is essential for stem cell migration and vascular development.^42^ Pro-angiogenic factors such as SDF-1α are essential to recruit endothelial cells to the wound bed and upregulate the expression of vascular endothelial growth factor.^43^ Additionally, our team also demonstrated the rejuvenating and anti-inflammatory potential of β-klotho on diabetic adipose derived stem cells and reportedly enhanced their pro-angiogenic potential.^41,44^ Klotho is endogenous transmembrane protein known for its anti-aging and anti-fibrotic roles by influencing the activity of fibroblasts growth factors.^45^ In our laboratory, we have previously shown i*n vitro* wound healing capabilities using a dual gene-activated collagen scaffolds containing both pro-angiogenic SDF-1α and anti-fibrotic β-klotho plasmids.

In the present study, by combining the regenerative capabilities of MSCs, we aimed to assess a novel anti-fibrotic, pro-angiogenic dual GAS (using the plasmids β-klotho and SDF-1α, respectively) on wound healing in aged Sprague Dawley rats when compared to the clinical standard gauze paraffin dressing Jelonet.

## II. RESULTS

### A. Assessment of wound healing—Macroscale observations

**FIG. 1.**
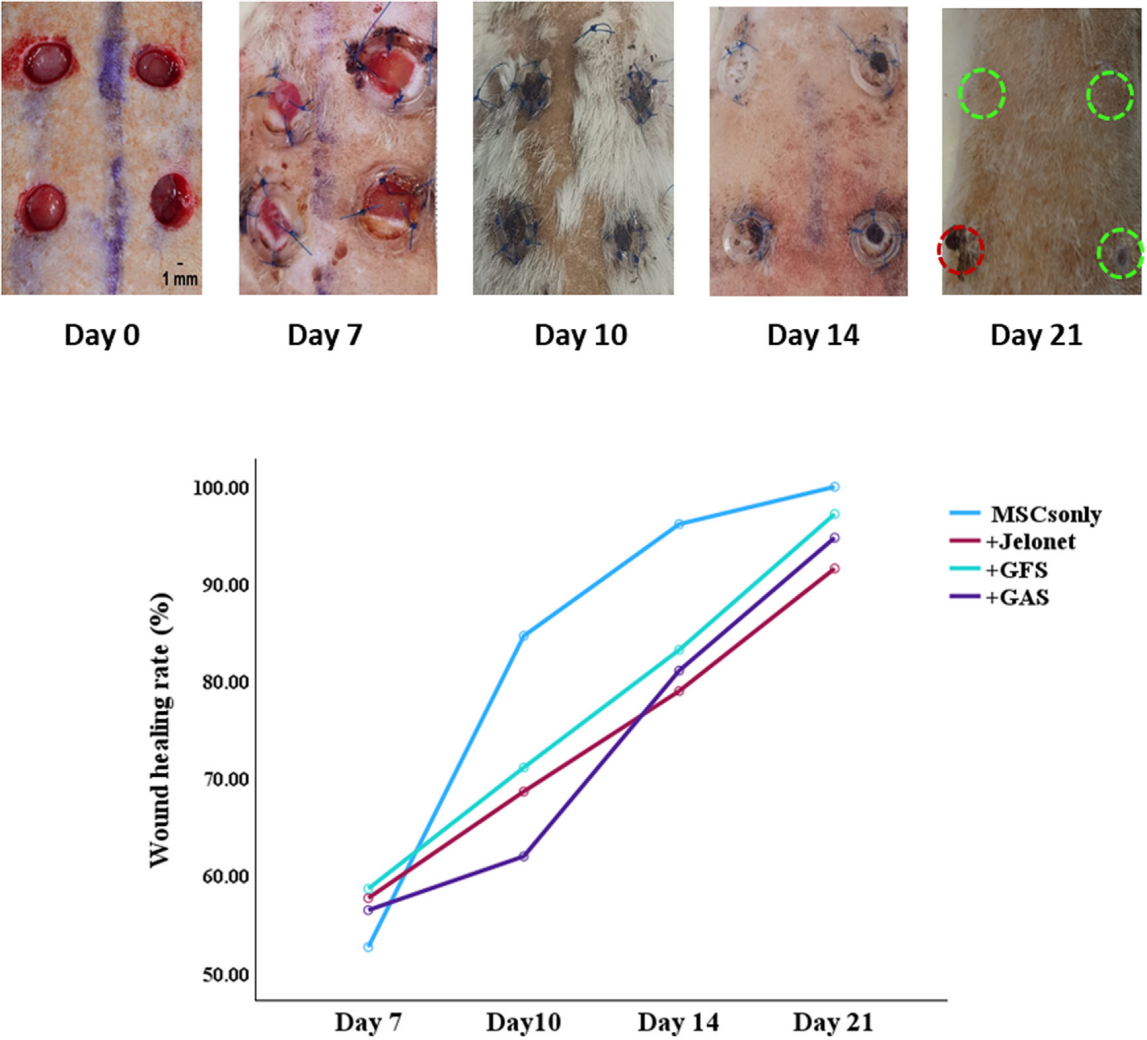
Wound size of rats at different time points post-surgery (day 0, day 7, day 10, day 14, and day 21). Complete wound closure is observed in “MSCs only” group at day 21, “+GFS” group showed 98% closure, and “+GAS” showed 95% closure, whereas “+Jelonet” group showed 92% closure.

There were no signs of pus in any groups during the study period, indicating there were no infections. The wound healing rates of different wound site conditions were evaluated in each animal. A gradual decrease in original wound size was shown over time in all studied groups (Fig. 1). The MSCs only group showed good closure from day 7 to day 21 (52.66 ± 1.70% to 100.0 ± 0.0%). The wound was significantly visible and was not completely closed at day 21 in the MSC +Jelonet group when compared to MSCs only group (p < 0.001), MSC +GFS (gene-free scaffold) (p = 0.003), and MSC +GAS gene-activated (p = 0.038) group (91.60 ± 1.30% vs 100.0 ± 0.0%, 97.2 ± 0.78%, and 94.7 ± 1.25%, respectively). +GFS group and +GAS exhibited similar appearance of wound closure with approximately 98% and 95% closure, respectively. A thick scab with exudate was also prominently seen over the +Jelonet group at day 21. The granulation tissue was fully covered, and the scab was detached in the MSCs only group by day 14 and in the +GFS and the +GAS groups by day 21 (Fig. 1).

### B. Assessment of wound healing—Histological evaluation

#### 1. Re-epithelization and epidermal thickness

**FIG. 2.**
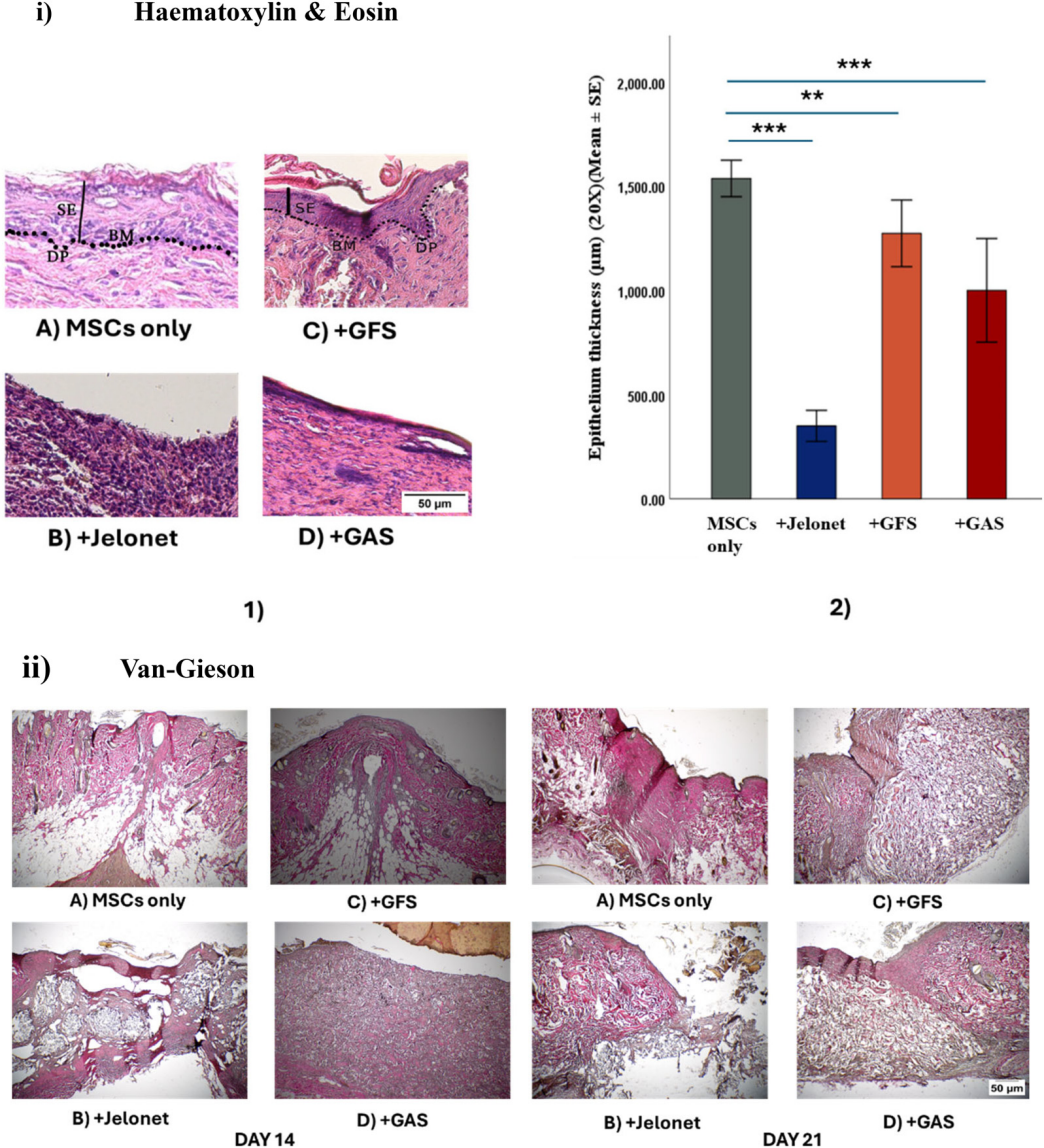

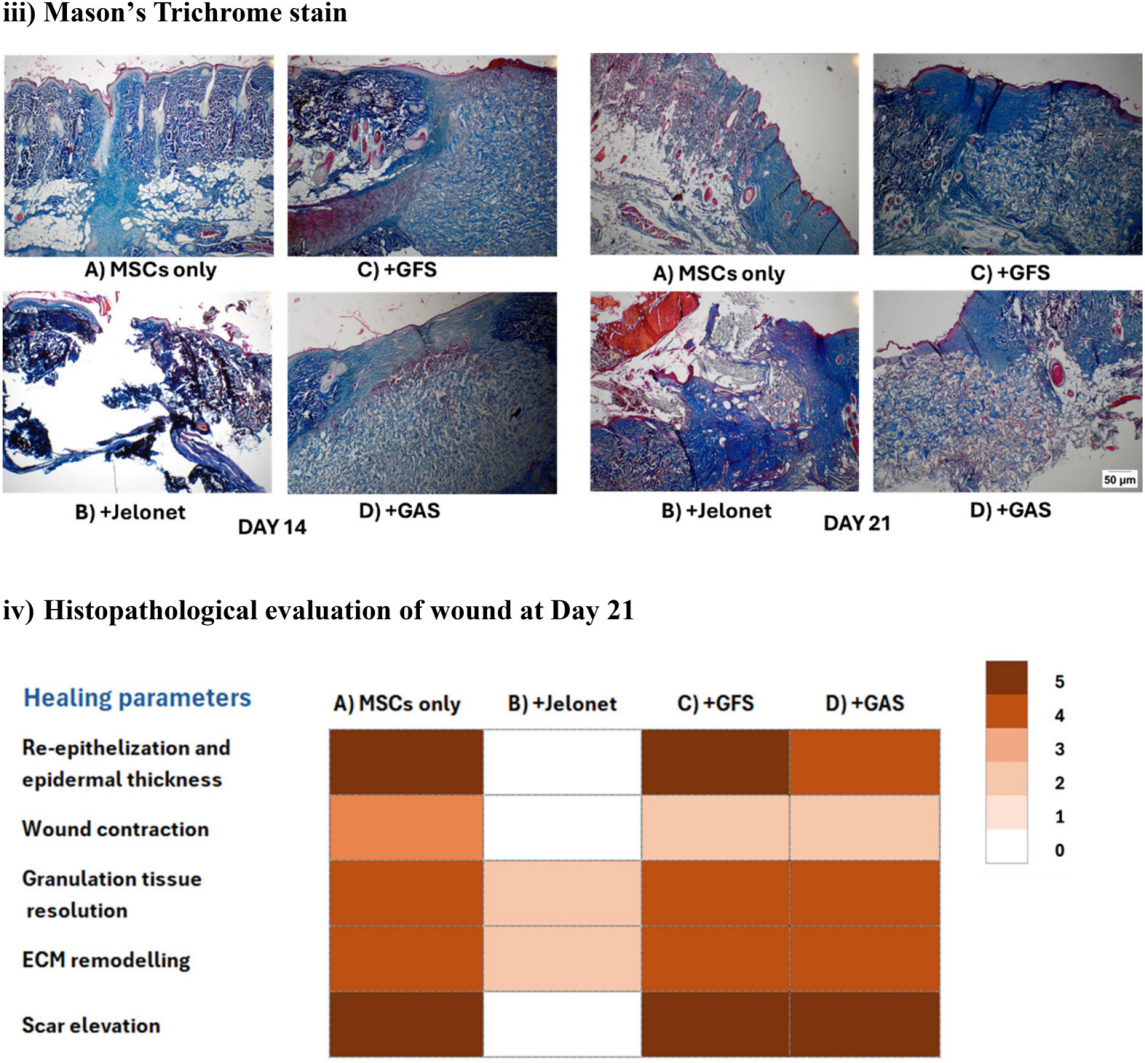
(i) H&E staining: (1) at day 21, stratified epithelium with stratum corneum, lucidum, granulosum, and spinosum is prominent in MSCs only and +GFS, SE: stratified epithelium, BM: basement membrane, and DP: dermal papillae; and (2) quantitative data of epithelial thickness (^***^denotes p < 0.001 and ^**^denotes p < 0.01). (ii) Van-Gieson staining shows the ECM collagen layout (pink to red stain) in the wound sites. (iii) Masson's trichrome staining shows that the ECM layout (collagen stained blue) in “+GFS” and “+GAS” groups is less condensed as compared to “MSCs only” group at day 21. (iv) Histopathological evaluation of various wound healing parameters scaled in a range 0–5.

H&E and Masson's trichrome staining revealed epithelial restoration by day 21 in MSCs only group, +GFS group, and +GAS group. The MSCs only group and +GFS groups were revealed to have restored all four layers of the stratified epithelium (stratum corneum, lucidum, granulosum, and spinosum), melanocytes, and the basal cells. Identical to MSCs only group, dermal papillae were evident in +GFS group as well [Fig. 2(i)]. The MSCs only group and +GFS groups had comparatively greater epithelial thickness as compared to +GAS and +Jelonet groups [Figs. 2(i-1) and 2(i-2)].

#### 2. Wound contraction

Histological analysis revealed distinct patterns of wound contraction across the experimental groups. MSCs only group exhibited pronounced contraction in the dermal and subcutaneous layer, while +GFS and +GAS groups showed less contraction [Fig. 2(ii)]. In +Jelonet group, the absence of wound closure precluded any observable contraction.

#### 3. Granulation tissue maturation

Histological examination at day 21 revealed persistent and substantial infiltration of inflammatory cells, predominantly lymphocytes and eosinophils, in +Jelonet group. In contrast, in MSCs only, GFS and +GAS groups demonstrated resolution of granulation tissue by day 21 [Fig. 2(i)].

#### 4. ECM remodeling

Distinct patterns of collagen deposition were seen across the experimental groups. +GFS and +GAS groups exhibited uniform collagen deposition as compared to MSCs only group with condensed collagen deposition, while +Jelonet group displayed disorganized collagen deposition, indicating an underdeveloped dermis [Figs. 2(ii) and 2(iii)].

#### 5. Scar elevation

Histological examination revealed that MSCs only, +GFS, and +GAS exhibited intact dermal layers with thicknesses comparable to that of uninjured skin by day 21. In contrast, +Jelonet displayed altered dermal integrity and thickness, deviating from the normal dermal architecture [Fig. 2(iii)].

A comprehensive histological assessment of wound healing parameters was conducted on day 21 post-injury. The evaluation involved grading various wound healing parameters and is summarized in Fig. 2(iv).

### C. Immunofluorescence assessment of wound healing

#### 1. Angiogenesis and cellular proliferation

**FIG. 3.**
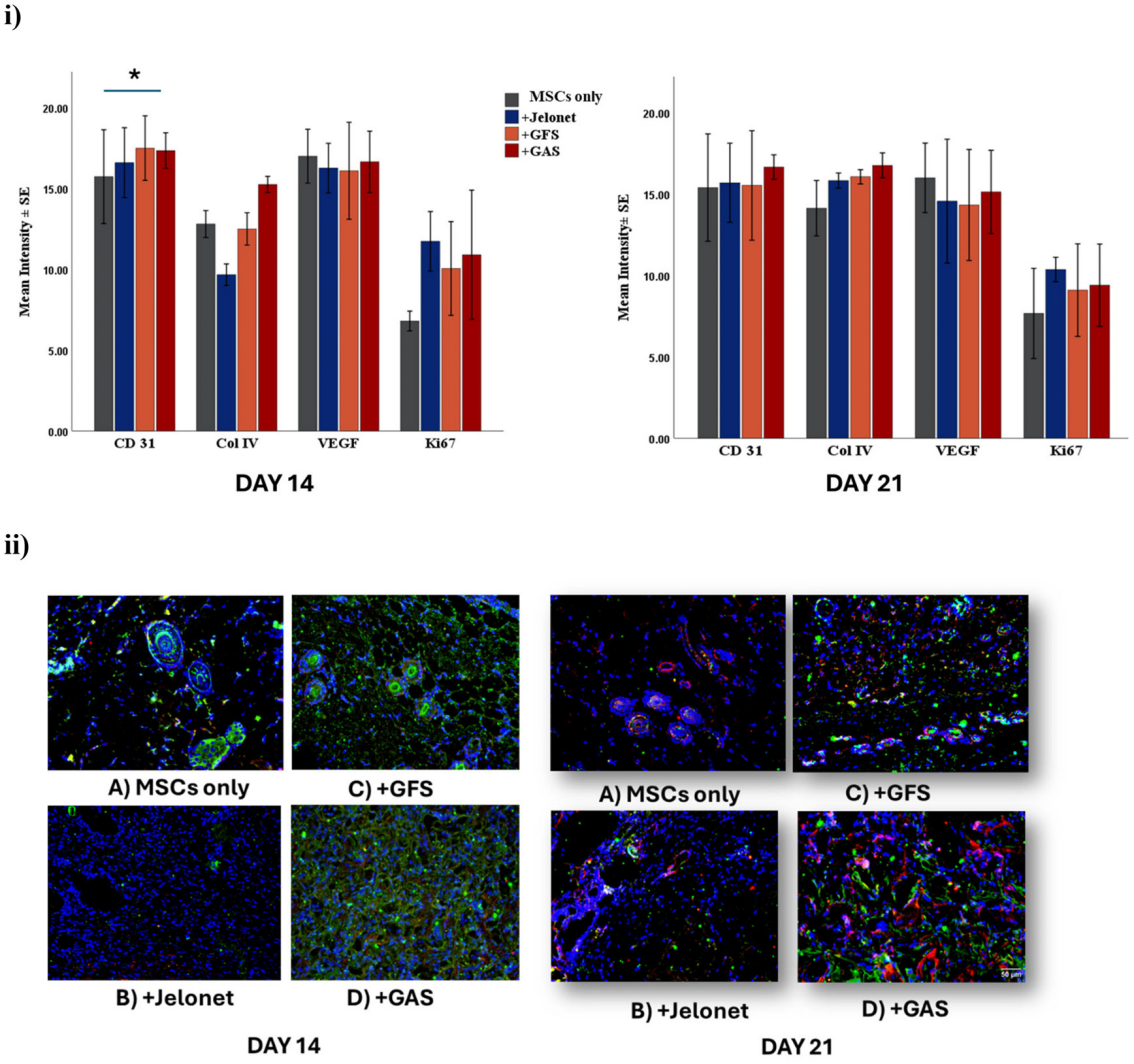
(i) Expression of CD31 was higher in +GAS group as compared to other groups both at day 14 and day 21 (p = 0.025). Cell proliferation marker Ki67 was higher in all the groups when compared to MSCs only group both at day 14 and day 21. (ii) Merged images of immunoflurosence staining of CD31 (green), Col IV (red) with DAPI (blue) in all four groups at day 14 and day 21.

Neovasculature staining using CD31 and Col IV complement each other. Immunofluorescence analysis revealed that CD31 was highest in +GAS groups compared to other groups (p = 0.025) at day 14. Post hoc analysis revealed that CD31 was higher in +GAS compared to MSCs only group at day 14 (p = 0.03). Expression of CD31 was still higher in +GAS group compared to other groups at day 21; however, this was not significant [Fig. 3(i)]. The basal lamina of all the capillaries were stained well with Col IV as wound healing proceeded, with the highest intesity of CD31 and Col IV in +GAS group, followed by +GFS group at day 21 [Fig. 3(ii)].

Expression of VEGF did not differ significantly across the groups at day 14 and day 21. Cellular proliferation was higher in +Jelonet, +GFS, and +GAS groups as compared to MSCs group; however, the finding was not significant.

#### 2. Modulation of ECM proteins

**FIG. 4.**
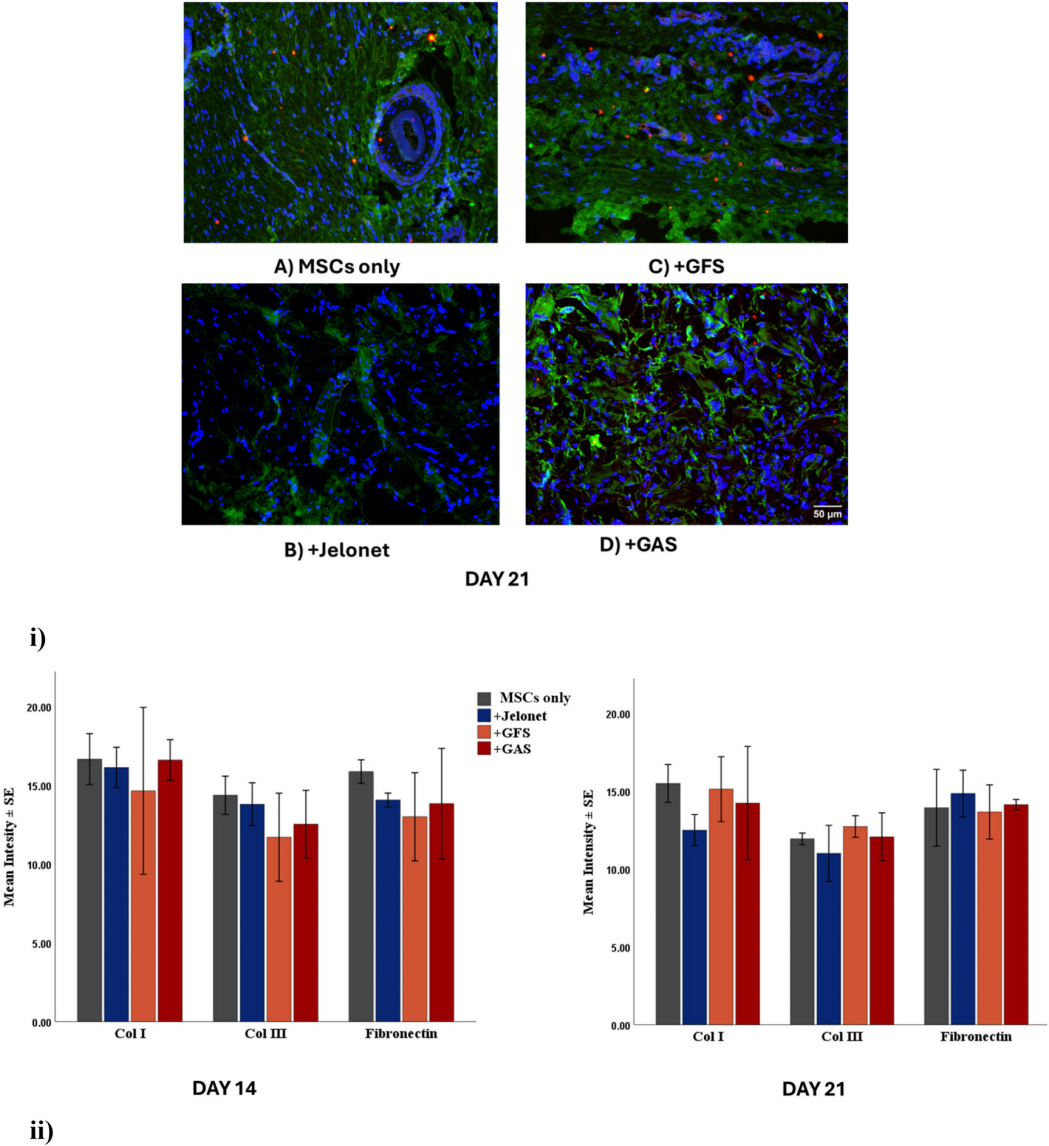
(i) Merged images of immunoflurosence staining of Col I (green), Col III (red) with DAPI (blue) in all four groups at day 21. (ii) The changes in ECM proteins in the four groups at day 14 and day 21.

ECM deposition during tissue repair determines wound scarring. Total relative collagen content did not differ significanlty across the groups from day 14 to day 21. Expression of Col III decreased in all the groups as the wound healing proceeded (day 21) [Figs. 4(i) and 4(ii)]. Expression of fibronectin decreased in MSCs group from day 14 to day 21, and its expression was at par in all other groups both at day 14 and day 21 [Fig. 4(ii)].

#### 3. Inflammatory response and M2 macrophage polarization

**FIG. 5.**
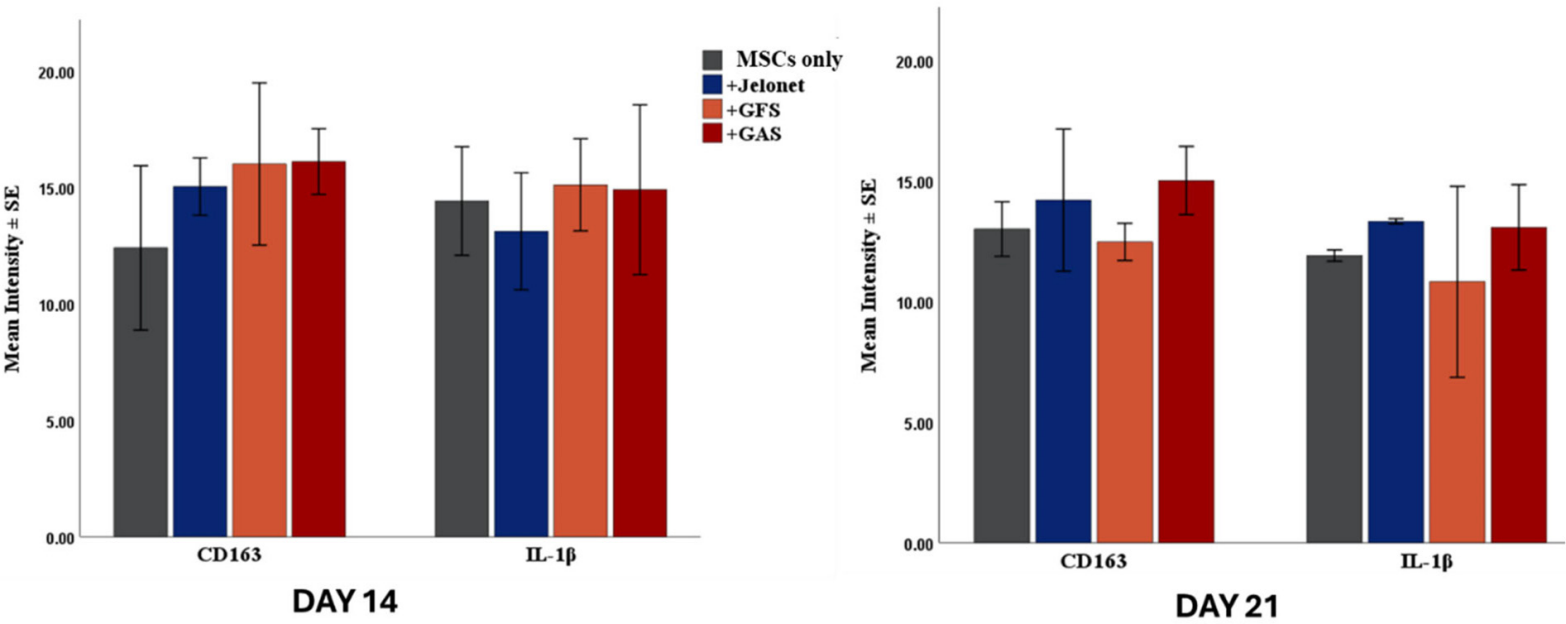
Expression of CD163 was not significantly different across the groups at day 14 and day 21. IL-1β decreases in the MSCs only and +GFS groups from day 14 to day 21. The decrease in IL-1β from day 14 to day 21 is significant in +GFS group (two way ANOVA, p = 0.007).

Resolving inflammatory phase is an important step in wound healing. IL-1β, a pro-inflammatory cytokine, is noted to decrease in the MSCs only and +GFS group from day 14 to day 21; +Jelonet and +GAS groups exhibited higher levels of IL-1 β till day 21 (Fig. 5). The decrease in IL-1β in +GFS group from day 14 to day 21 was significant when assessed with general linear model for two way ANOVA (p = 0.007).

M2 polarization is essential to transiently swtich the pro-inflammatory enviroment to anti-inflammatory environment to proceed to proliferative phase of wound healing. CD163 (a surface marker for M2 macrophage) was expressed more in +Jelonet, +GFS, and +GAS groups at day 14 (the difference was not siginificant on one-way ANOVA). However, by day 21, the expression levels of CD163 were comparable among all four groups.

## III. DISCUSSION

There have been many tissue engineering approaches to treat nonunion skin ulcers using cells, scaffolds, and bioactive molecules. With the complexity of aging, senescence, impairing wound healing, and a global increasing aging population, the rate of chronic skin wounds is increasing. Similarly, increasing is the need for novel age specific wound healing approaches.

In this study, we examined cutaneous skin wound healing in aged skin of 2-year-old Sprague Dawley male rats. We compared the response of MSC cell therapy alone or with the clinical standard paraffin dressing (+Jelonet). These were compared against MSCs seeded on collagen sponges CG-GAG (+GFS) without or with gene-activation (+GAS). These constructs were implanted in a split skin wound model, and wound healing was monitored for 21 days.

The findings from this study revealed differences in healing patterns between these groups. In this study, the MSCs only group showed 96.1% wound closure in the aged rats at day 14 with complete re-epithelization by day 21, while another study on the brown Norway rats aged 17 months old demonstrated ∼70%–75% wound closure at day14.^46^ The observed mild to moderate inflammation aligns with the established characteristics, as bone marrow derived MSCs are known for their hypo-immunogenic properties, and hence lower rejection/immune reaction has been reported with transplantation of allogenic MSCs.^47,48^ The rapid closure of the wound site could be attributed to the role of MSCs in promoting angiogenesis and being less immunogenic.^49^ The histological evaluation of the wounds indicated mild to moderate mixed inflammation and granulation along with notable abrupt wound contraction and deep mid fibrosis in the MSCs only group at day 14. This was expected in a split skin model of healthy animals albeit aged but not using a critical sized defect. However, in the scaffold groups (MSCs + GFS and MSCs + GAS), this level of wound contraction was not observed overtime. The collagen–glycosaminoglycan scaffolds (Collagen–GAG) has been used widely and has been reported to induce tissue regeneration by acting as a matrix for enhanced cell adherence and proliferation, thereby reducing wound contraction and scar formation.^50^ These findings indicate that the incorporation of MSCs within the gene-free and gene-activated scaffold facilitated a more uniform transient expression of their regenerative potential. This synergistic effect of MSCs and the +GFS resulted in a more efficient and organized healing process, potentially due to the scaffold's ability to provide a supportive microenvironment for MSCs for enhanced host cell adhesion and migration, promoting enhanced remodeling with sustained and structured wound healing.^51–53^

Similarly, in the Jelonet group, the wound did not contract maintaining the wound in a hydrated state. The +Jelonet group with MSCs failed to show complete epithelization at day 21 with only 92% wound closure. Histology of the +Jelonet wound section showed skin ulceration, granulation tissue, and foreign body giant cell reaction at day 14. The results of the +Jelonet group in this study are similar to other studies where they concluded that using occlusive dressing specifically Jelonet promotes the granulation tissue formation over re-epithelization.^14,54^

The growth factors and the cytokines released by MSCs can modulate the wound bed to a more pro-angiogenic state. It is essential that the granulation tissue formed is vascularized as the wound healing progresses.^55^ Earlier studies with topical application of SDF-1α on scaffold in split skin wound models have shown similar results with reduced wound contraction and reepithelization; however, neovascularization assessments were not explored well enough nor *in vivo* with aged skin.^56^ CD31 or PECAM-1 is marker for angiogenesis, and in this study, we demonstrated that the +GAS group had significantly higher expression of CD31 at day 14 and at day 21 as compared to MSCs only group, indicating that the +GAS (SDF-1α and β-klotho) could induce neovascularization and enhanced angiogenesis. It was interesting to note the expression of CD31 synergized with that of Col IV in all groups with the highest expression in +GAS group. Col IV acts as a key structural element of the basement membrane, essential for maintaining the integrity and stability of capillary structures, thereby indicating that the enhanced expression relates to the formations of firm and matured basement membrane of the vessels. Combining the fluorescence of CD31 with Col IV can give us a clear picture of the complete range of neo-vessels formed during wound healing.^57,58^

The +GAS group enhanced wound healing by slow release of the targeted growth factor and programming the matrix making it a depot of localized expression for the wound bed similar to previously described by Deshane *et al.*^43^ Enhancing vascularization in scaffold is a crucial parameter determining the success of tissue engineering strategies and may be the reason for a better healing response in MSC+ GAS vs the MSC +Jelonet, a wound dressing substitute used for chronic wounds in clinical setting.^41,59^

ECM layout and remodeling is one of the important milestones in wound healing. Col III is initially expressed and subsequently replaced by Col I as healing progresses.^60^ Our results showed a similar pattern with reduction in Col III by day 21 in all the groups. Fibronectin is another ECM protein essential for cell attachment and migration as wound healing proceeds. Overproduction of Col I and fibronectin are reported in hypertrophic scars and keloids.^61^ Our results showed that expression of Col I and fibronectin were uniform in all groups in this study.

It is worth noting that our study design focused solely on healthy aged wounds as a variable and did not assess for use of disease or infection models important in clinical setting like diabetic foot ulcers. The limitations of sample size to run a study with aged animals, housing them for 20–24 months, represent disadvantages of age-related animal-based research. Although the surgeons were blinded, about the groups, the study investigators were not blinded. In addition, randomization of the treatment groups would have reduced any potential bias and further enhanced the rigor and reproducibility of the study findings.

Chronic wound treatment is an ongoing medical burden. In the light of an increasing aging population, it is imperative to find new avenues using a holistic approach, where tissue engineering methods hold great prospects. It is quite intuitive that the clinical decision to choose the best treatment modality for each wound milieu should be evidence-based.^36^ The evidence presented in this study indicates that utilizing tissue engineering aspects, MSCs in gene-free scaffolds support integrative wound healing, and this could be enhanced with the addition of (pro-angiogenic SDF-1α and anti-fibrotic β-klotho) gene-activated scaffolds. We find that MSCs alone showed the greatest contraction, and the clinically used gauze Jelonet gave the least healing. These results show that the scaffolds not only have the ability to support skin wound healing but also may be engineered using programmed gene-activation to direct wound healing ability of MSCs. This may be particularly important in treating complex wounds that require cellular activation for enhanced angiogenesis and reduced fibrosis like in diabetic foot ulcers. Engineering these scaffolds with stem cells and therapeutic stimulants hold potential in tissue engineering and chronic wound healing.^62,63^

## IV. CONCLUSION

Aging is associated with cellular senescence and failures in biomolecular repair mechanisms which may eventually lead to chronic skin wounds. In this study, a MSC seeded split skin wound model was used to assess biomaterial wound healing in 2-year-old aged Sprague Dawley rats.

We report that the clinical standard Jelonet gauze with MSCs displayed the least wound healing, no epithelization or tissue integration, and only exudate with scab formation by 21 days. However, MSCs combined with collagen GAG scaffolds (gene-free) showed tissue integration, intact epidermal epithelization, and wound healing at 21 days. Neo-vascularization of the wound bed could be enhanced by a pro-angiogenic SDF-1 α plasmid and fibrosis restricted using an anti-fibrotic β-klotho plasmids in the form of a MSC loaded gene-activated scaffold. Both gene-free and gene-activated scaffold groups demonstrated unified healing with tissue integration and reduced contraction when compared with MSCs only and Jelonet. Our findings indicate that dual gene-activated collagen scaffolds combined with MSCs can modulate wound healing in aged rats and may be useful to heal chronic wounds such as diabetic foot ulcers where enhanced angiogenesis and limited fibrosis are needed. These findings underscore the potential synergistic effects of combining MSCs with biocompatible support scaffolds, and they can be enhanced with programmed gene-activation to direct a controlled wound healing in age-related skin regeneration.

## V. METHODS

### A. Preparation of gene-free collagen–GAG scaffold (GFS) and gene-activated scaffold (GAS)

GAS was made using a two-step procedure as defined previously.^44,64–66^ Optimized freeze-drying techniques were used to prepare scaffolds from bovine tendon type 1 collagen (0.5% w/v) and shark cartilage chondroitin-6-sulfate (0.05% w/v) (Sigma, UK). The collagen–chondroitin slurry blended in acetic acid (0.05 M) was degassed and then treated at 105 °C under vacuum for sterilization and scaffold cross-linking.^65,66^ To further increase the mechanical stability, the scaffolds were further chemically cross-linked with 14 mM N-(3-Dimethylaminopropyl)-N′-ethylcarbodiimide hydrochloride and 5.5 mM N-Hydroxysuccinimide (EDC/NHS, Sigma, UK) solution. Once the cross-linked scaffolds were ready, they were washed with PBS (Gibco, UK), and polyplex nanoparticles were loaded onto the scaffolds which were kept undisturbed for 40 min. As described in our previous studies, to develop a polyplex particle with a N/P ratio of 10, specific amount of branched cationic 25 kDa polyethyleneimine (PEI) (Sigma-Aldrich, Dublin, Ireland) was mixed for anionic pDNA (dose of 2 *μ*g).^44,64,67^ Cylindrical scaffolds (8 mm diameter and 4 mm in height) were used, and two scaffold groups were prepared: (1) gene-free scaffold (GFS, no pDNA), and (2) dual gene-activated scaffold (GAS) which contained SDF-1α and β-klotho plasmid complexed PEI nanoparticles (plasmids were obtained from SantaCruz Biotechnologies, USA and SinoBiological Beijing, China, respectively).

### B. Isolation and seeding of bone marrow derived mesenchymal stromal cells (MSCs)

Bone marrow derived mesenchymal stromal cells (MSCs) were isolated from young Sprague Dawley male rats (10–12 weeks) as per the protocol described by Nauta *et al.* and Yusop *et al.*^68,69^ Rats' femurs and tibias were dissected aseptically, and their ends were trimmed to expose the interior marrow shaft. The bone marrow was flushed with complete media by inserting a 23-gauge needle attached to a 5 ml syringe into a 50 ml falcon tube. Flushed marrow was then resuspended and passed through a 70-*μ*m filter mesh in a new falcon tube to remove any debris. The cell suspension was centrifuged at 1200 RPM for 5 min, and the cell pellet was resuspended in complete media Dulbecco's Modified Eagles Medium (DMEM) (Lonza, Belgium) supplemented with 10% fetal bovine serum (Thermo Fisher Scientific, Waltham, MA) and 1% penicillin streptomycin (Thermo Fisher Scientific, Waltham, MA). After plating in a T-25 flask, the cultures were incubated at 37 °C in a humidified atmosphere containing 5% CO_2_.

The tissue culture medium was changed after 48 h, and the nonadherent cells were removed, then changed every 2–3 days. When adherent cells reached 80% confluence level, they were washed twice with phosphate buffer saline (PBS) and detached with 0.25% trypsin/ethylenediamine tetra-acetic acid (EDTA) (Thermo Fisher Scientific, Waltham, MA) solution. The cells were split at a ratio of 1:3. MSCs at passage three (P3) were used for the current experiments. Follow-up of cultured cells was done using a phase-contrast inverted microscope equipped with a digital camera (IX73 Olympus, Japan).

### C. Animal handling

The study was approved by the RCSI Bahrain Ethics Committee REC/2020/41 and Bahrain Defense Forces hospital ethics no. BDF/R&REC/2021-558. All the animals were handled as per international animal welfare standards. To ensure reliable identification, each animal was tagged with a microchip. These microchips were implanted subcutaneously in the inter-scapular region using a minimally invasive procedure.^70^ Animals were inspected by a veterinarian and underwent a 10-day isolation period before joining the animal holding unit. All the animals were caged separately in a room maintained at a temperature of 22 ± 1 °C, with a humidity of 50%–60% and 12/12 h of light/dark cycle.

### D. Wound induction and surgery

The surgical procedure was conducted in accordance with ethical standards and protocols. Fourteen male Sprague Dawley rats, aged 20–24 months, were utilized in this study. Anesthesia was induced using isoflurane (4%–5% in a gas chamber for induction and 2%–3% for maintenance), delivered via a nose cone throughout the procedure. The dorsal region of the animals was shaved and disinfected with antiseptic (10% povidone-iodine). The animals were then placed in a prone position on pre-warmed heat pads to maintain thermoregulation.

**FIG. 6.**
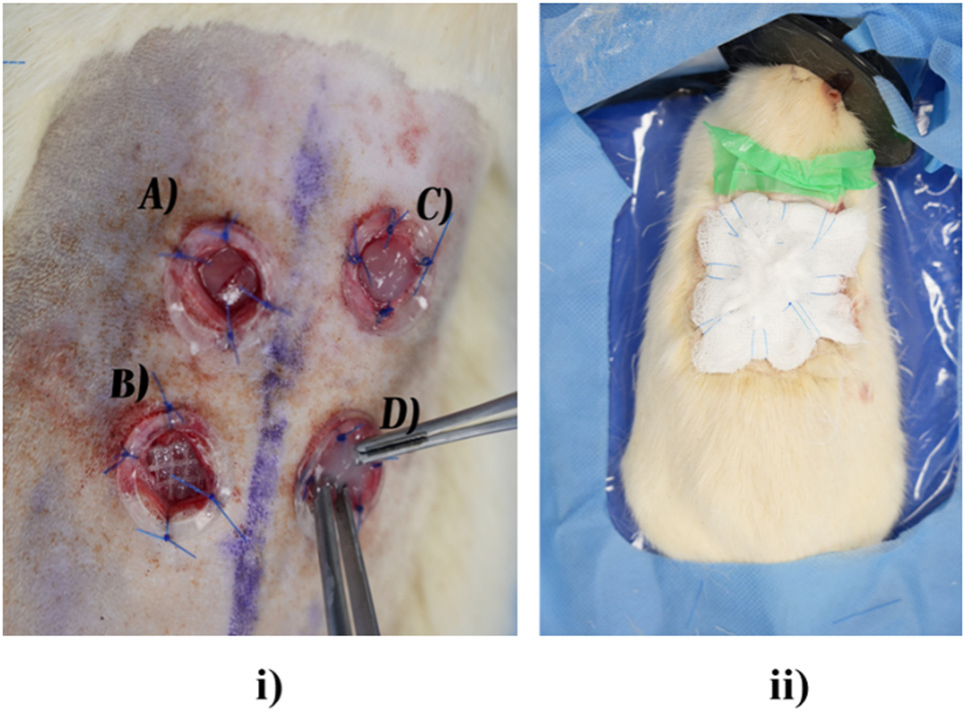
(i) Four full-thickness split skin defects of aged rats with (a) upper left site with MSCs only, (b) lower left site has MSCs with Jelonet, (c) upper right is GFS seeded with MSCs, and (d) lower right is GAS with MSCs. Wound splints are sutured around the wound; (ii) postsurgical dressing with Opsite and dry gauze using six-loop dressing technique.

Using a sterile biopsy punch, four 8-mm full-thickness circular wound biopsies were made bilaterally on the dorsal surface of the rats' skin (two on the left and two on the right of the dorsal back), ensuring that the wounds were at least 1.5 cm apart. The skin flaps were then excised using iris scissors. After excision, circular silicone splints with an inner diameter of 10 mm were placed on top of the wounds and sutured into position using 3-0 nylon sutures (Fig. 6). The following biomaterials and cells were loaded into the wounds: (A) upper left quadrant allogenic mesenchymal MSCs only group were seeded with 50 *μ*L of 2 × 10^6^ rat MSCs; (B) lower left quadrant Jelonet gauze seeded with 50 *μ*L of 2 × 10^6^ rat MSCs (+Jelonet); (C) upper right quadrant contained collagen glycosaminoglycan scaffold seeded with 50 *μ*L of 2 × 10^6^ rat MSCs (gene-free scaffold or GFS group); and (D) lower right quadrant gene-activated (SDF-1α and β-klotho) soak loaded onto collagen–glycosaminoglycan scaffold seeded with 50 *μ*L of 2 × 10^6^ rat MSCs (gene-activated scaffold or GAS group).

Following the surgery and the loading of the assigned cells and biomaterials, the wounds were covered with Opsite and dry gauze. To prevent displacement, loops were created around the gauze and secured together. These loops remained intact, serving as a framework for future gauze replacement and wound dressing changes for intended wound assessments. The animals were then placed in an incubator at 30 °C until recovery and were later caged separately to avoid cross-infections. They were monitored daily for 21 days.

The team successfully developed a standard surgical and postsurgical protocol. The six-loop dressing technique was effective in keeping the scaffolds securely in position throughout the study.

### E. Evaluation of wound healing

Skin wounds were photographed using a digital camera, and the wounds' surface area was measured using a measuring scale for all rats on day 7, 10, 14, and 21 (re-epithelization or complete closure of the wound). The duration taken for wound closure was recorded for statistical comparison. Wound shrinkage rate was calculated as [(original area − epithelized wound area)/original area)] × 100%.

### F. Histological evaluation—H&E, Van Gieson, and Masson's trichrome staining

The treated animals were euthanized at day 21, and skin tissues were collected. Skin biopsies were obtained using a sharp scalpel (including the granulation tissue formed at the center and the normal surrounding skin at the periphery). Skin tissue was fixed overnight in 4% paraformaldehyde at 4 °C, processed using an automated tissue processor (Leica, Nusslock, Germany), and then embedded in paraffin. 5 *μ*m thick sections were cut using a rotary microtome and were mounted on L-polysine coated glass slides (Sigma-Aldrich, France). Samples were deparaffinized and then rehydrated in alcohol prior to staining with hematoxylin & eosin (H&E) (Biognost, EU), Van Gieson (Abcam, UK), or Masson's trichrome (Abcam, UK). Images were acquired by cellSens Imaging Software (Evident, Olympus Life Science Solutions, Japan) at X 4, 10, and 20 magnifications.

A qualitative evaluation of histological parameters indicative of wound healing progression was conducted based on criteria described by Arslantas *et al.* and Van De Vyver *et al.*,^71,72^ and histopathological score to assess wound healing was formulated (Table I).

**TABLE I.** Histopathologic score to assess wound healing.

	0	1	2	3	4	5
Re-epithelization and epidermal thickness	Absent	Minimal	Partial	Moderate	Nearly complete	Complete and mature
Wound contraction	Excessive (considered detrimental)	Severe	Moderate	Mild	Minimal	Optimal (balanced contraction)
Granulation tissue maturation	Immature	Minimal maturation	Mild maturation	Moderate maturation	Advanced maturation	Fully matured
ECM remodeling	Absent	Minimal, disorganized	Mild, slightly organized	Moderate, partially organized	Abundant, mostly organized	Extensive, well-organized
Scar elevation	Mild hyperplasia	Minimal hypoplasia	Moderate hypoplasia	Mild hypertrophied	Moderate hypertrophied	Normal

### G. Immunofluorescence investigations

Paraffin embedded tissue was sectioned into 5 *μ*m thick sections and mounted on L-polysine coated glass slides (Sigma-Aldrich, France). The sections were deparaffinized, then permeabilized with 0.2% Tween^®^20 (Sigma-Aldrich, France) solution in PBS for 30 min (10 min wash × 3), and blocked using 10% NGS (Normal Goat Serum, Invitrogen, UK)/5% BSA/0.3 M Glycine (prepared in permeabilizing solution) for 1 h. Antibodies against following markers were allowed to react overnight at 4 °C: Ki67 (1:200), collagen IV [Col IV] (1:100), CD31 (1:100), VEGF-A (1:500), fibronectin (1:200), CD163 (1:100), IL-1 β (1:200), collagen I [Col I] (1:200), and collagen III [Col III] (1:500) (Table II).

**TABLE II.** List of antibodies.

Indicators	Primary antibodies (Catalog no.)	Dilutions in 1% BSA solution
Angiogenesis	CD31 (ab119339, Abcam, UK)	1:100
	VEGF-A (ab1316, Abcam, UK)	1:500
ECM proteins	Collagen I (NB600-450, Novusbio, UK)	1:200
	Collagen III (NB600-594, Novusbio, UK)	1:500
	Collagen IV (ab6586, Abcam, UK)	1:100
	Fibronectin (ab2413, Abcam, UK)	1:200
Cell proliferation	Ki-67 (ab16667, Abcam, UK)	1:200
Inflammation	IL-1 β (ab156791, Abcam, UK)	1:200
M2 macrophage	CD163 (ab156769, Abcam, UK)	1:100

After primary antibody incubation, the slides were rinsed by PBS and then covered in either Alexa 488-conjugated goat anti-mouse IgG (Cat no. A32723, Invitrogen, UK) and/or Alexa 594-conjugated goat anti-rabbit IgG (Cat no. A11012, Invitrogen, UK) at 1:800 dilution at room temperature for 1 h in the dark. The rinsing steps were performed as previously and counterstained for nuclei using the mounting medium with DAPI (ab104139, Abcam, UK). The images were taken under a fluorescence microscope (Olympus BX43, Japan) at 20× magnification. Samples were incubated with only secondary antibodies as controls.

Images were taken to capture all the changes from epidermal to dermal layer of the healed tissue using cellSens Imaging Software (Evident, Olympus Life Science Solutions, Japan) and semi-qualified by ImageJ. Cell counting was performed on the images using ImageJ software. The background and exposure parameters were calibrated using a negative control. Background-subtracted fluorescence intensity measurements were normalized to cell density, and the results were presented as mean ± standard error of the mean (SEM).

### H. Statistical analysis

General linear model of ANOVA and Bonferroni post hoc tests were used for multiple comparisons. Statistical analysis was done using SPSS statistical software (SPSS 26.0, SPSS, Inc., Chicago, IL, USA). A p value of <0.05 was considered statistically significant.

## Data Availability

The data that support the findings of this study are available from the corresponding author upon reasonable request.
